# Metabolism-Relevant Molecular Classification Identifies Tumor Immune Microenvironment Characterization and Immunotherapeutic Effect in Cervical Cancer

**DOI:** 10.3389/fmolb.2021.624951

**Published:** 2021-07-01

**Authors:** Luyi Li, Hui Gao, Danhan Wang, Hao Jiang, Hongzhu Wang, Jiajian Yu, Xin Jiang, Changjiang Huang

**Affiliations:** ^1^ Institude of Environmental Safety and Human Health, Wenzhou Medical University, Wenzhou, China; ^2^ Key Laboratory of Fertility Preservation and Maintenance of Ministry of Education, Ningxia Medical University, Yinchuan, China; ^3^ The 2^nd^ Afflicated Hospital and Yuying Children’s Hospital, Wenzhou Medical University, Wenzhou, China; ^4^ Prenatal Diagnosis Center of NanFang Hospital, The Southern Medical University, Guangzhou, China

**Keywords:** cervical cancer, classification, metabolism, immunity, immunotherapy

## Abstract

Cervical cancer (CESC) is a gynecologic malignant tumor associated with high incidence and mortality rates because of its distinctive management complexity. Herein, we characterized the molecular features of CESC based on the metabolic gene expression profile by establishing a novel classification system and a scoring system termed as METAscore. Integrative analysis was performed on human CESC samples from TCGA dataset. Unsupervised clustering of RNA sequencing data on 2,752 formerly described metabolic genes identified three METAclusters. These METAclusters for overall survival time, immune characteristics, metabolic features, transcriptome features, and immunotherapeutic effectiveness existed distinct differences. Then we analyzed 207 DEGs among the three METAclusters and as well identified three geneclusters. Correspondingly, these three geneclusters also differently expressed among the aforementioned features, supporting the reliability of the metabolism-relevant molecular classification. Finally METAscore was constructed which emerged as an independent prognostic biomarker, related to CESC transcriptome features, metabolic features, immune characteristics, and linked to the sensitivity of immunotherapy for individual patient. These findings depicted a new classification and a scoring system in CESC based on the metabolic pattern, thereby furthering the understanding of CESC genetic signatures and aiding in the prediction of the effectiveness to anticancer immunotherapies.

## Introduction

Cervical cancer, which classified into two histological subtypes, namely cervical squamous cell carcinoma and endocervical adenocarcinoma (CESC), is the 4^th^ prevalent malignant tumor worldwide (Miller et al., 2019). According to GLOBOCAN statistics, in CESC, there are approximately over 530,000 new cases and 260,000 deaths annually, and the morbidity accounts for 73–93% of all types of female gynecologic malignant morbidity. In China alone, over 130,000 cases are diagnosed annually (Song et al., 2017; Bray et al., 2018; Jassim et al., 2018). Despite the new diagnostic methods and clinical treatments for CESC emerge currently, its prognosis still remains dismal. Therefore, profound insights into the mechanisms underlying CESC genetic diversity at molecular level are needed for the development of precise diagnosis and personalized therapies. Recently, genome-wide mRNA expression patterns analyses have been proved valuable for this purpose. Yet, the relationships between the molecules and the clinicopathology of CESC have not been comprehensively investigated.

Cancer is believed as a metabolic-disorder disease (Coller, 2014; Boroughs and DeBerardinis, 2015). Many cancer mutations and cancer-related genes interfere with metabolic processes including one-carbon metabolism, erobic glycolysis and glutaminolysis which all support tumor cell growth and proliferation (Fiehn et al., 2016). With respect to CESC, it as well shows the correlation between the intratumoral metabolism and gene mutation heterogeneities (Kidd and Grigsby, 2008; Penaranda et al., 2013; Li et al., 2017; Shu et al., 2020). It has been discovered that glycolytic cervical tumor cells existed in a relative state of oxidative stress due to the increased reactive oxygen species levels, and was compensated by upregulating redox metabolic pathways (Schwarz, 2019). Besides, the metabolic changes including obesity, elevated blood pressure and triglycerides presented in the metabolic syndrome (MetS) have been confirmed the association with the incidence of CESC (Kidd and Grigsby, 2008; Ulmer et al., 2012; Penaranda et al., 2013). Furthermore, a retrospective study has verified that MetS including impaired fasting glucose and hypertriglyceridemia was related to higher recurrence risk in early-stage CESC patients (Ahn et al., 2015).

More interestingly, mounting evidence has been publicized that the plasticity of immune function occurred in distinct metabolic signatures (Domblides et al., 2018). Some studies have shed light on modulating immune function and polarization through targeting some particular metabolic patterns, consequently providing therapeutic potential for various immune-mediated disorders including cancer. In more depth, previous data has revealed that tumor microenvironment affected T cell metabolism which impacted T-cell response to tumors, offering a means of ameliorating the T cell response through metabolic manipulation which might improve the effectiveness of cancer immunotherapy (Dugnani et al., 2017). Together, these findings underscore the importance of analyzing the genetic landscape of CESC from the metabolic prospective. Accurate metabolic-relevant subpopulation identification and characterization are essential for comprehending this disease and allowing for maximum efficacy of immunotherapy.

Hence, in this study, CESC data downloaded from The Cancer Genome Atlas (TCGA) was identified three METAclusters based on 2,752 metabolic genes. Survival prognosis, immune characteristics, transcriptome features, metabolic features, and immune checkpoints expression in CESC METAclusters differed generally. Then 207 differentially expressed genes (DEGs) among three METAclusters were identified three geneclusters for internal validation. Finally, METAscore, a metabolism-scoring system, was determined as an independent prognostic biomarker, and its potential to predict immunotherapeutic effects was probed. In conclusion, a novel metabolism-related classification was generated, while, evaluation the metabolism pattern of individual patient would contribute to diagnose and guide more effective immunotherapy strategies.

## Materials and Methods

### Cervical Cancer Data Source and Preprocessing

Our study for publicly available CESC gene-expression data including 291 patients was yielded on TCGA, which downloaded from the UCSC Xena browser (GDC hub: https://gdc.xenahubs.net), and analyzed using the R software (version 3.6.1) and R Bioconductor packages.

### Clustering of Metabolism-Associated Genes in CESC

The unsupervised clustering method of assessed metabolic genes was employed to classify patients into multiple clusters for further assessment by using the ConsensusClusterPlus R package. Then the value for *k*, where the cophenetic correlation coefficient magnitude began to fall was selected as the optimal cluster number (Hartigan and Wong, 1979). This analysis has been confirmed the classification stability for repeating 1,000 times (Monti et al., 2003).

### Estimation of Immune Characteristics

The consensus ESTIMATE (Estimation of STromal and Immune cells in MAlignant Tumor tissues using Expression) algorithm with ESTIMATE R package was employed to measure ESTIMATE, immune and stromal scores, which reflected the immune and stromal cell gene signatures enrichment (Yoshihara et al., 2013).

Single-sample GSEA (ssGSEA) with GSVA R package was used for estimating immune infiltration in different clusters, and then an enrichment score indicated the extent to which genes were coordinately up or down-regulated within a single sample (Barbie et al., 2009).

### Differentially Expressed Genes (DEGs) Associated With METAclusters and Generated Geneclusters for Validation

Next, DEGs among the CESC METAclusters were identified using the R limma package. Only the genes with | logFC| > 1 (adjusted *p* < 0.01) were regarded as DEGs. Based on the above differential genes, genes with significant prognostic value were utilized for gene clustering by using the ConsensusClusterPlus R package.

### Metabolic-Based Prognostic Model Construction

Principal component analysis (PCA) was done and PC1 was selected as the signature score. After acquiring the prognostic value of each gene biosignature score, the following formula was used to describe the METAscore of every CESC patient:

METAscore = ∑PC1i-∑PC1j (1)

which *i* is the signature score of clusters with positive Cox coefficient, and *j* is the expression of genes with negative Cox coefficients.

### Functional and Pathway Enrichment Analysis

Gene set variation analysis (GSVA) is a unsupervised and nonparametric gene set enrichment approach that estimates biosignature scores or pathways based on transcriptomic data (Hänzelmann et al., 2013). We downloaded the gene sets from MSigDB (Broad Institute) (Subramanian et al., 2005), and chose gene ontology (GO) gene sets to quantify pathway activity. Pathway analysis was processed based on METAclusters and METAscore by using the GSVA R package in this study.

### Estimation of the Benefit of METAscroe for Immunotherapy

The TIDE (tumor immune dysfunction and exclusion) algorithm was applied to predict the potential response to immune checkpoint blockade (ICB) therapy of METAscore. For the melanoma dataset (GSE78220, *N* = 28), expression patterns (FPKM normalized) and phenotypes were transformed into transcripts per kilobase million (TPM), converting the FPKM data to values more comparable between samples to calculate METAscore (Wagner et al., 2012).

### Statistical Analysis

The normality of the variables was verified using the Shapiro-Wilk normality test (Ghasemi and Zahediasl, 2012). For comparisons between two groups, statistical significance was estimated using the unpaired Student t-tests and Wilcoxon tests for normally distributed variables and non-normally distributed variables, respectively. For comparisons between more than two groups, Kruskal-Wallis tests and one-way analysis of variance (ANOVA) were employed as nonparametric and parametric techniques, respectively (Hazra and Gogtay, 2016). Pearson and distance correlation analyses were conducted for correlation coefficients. Two-sided Fisher exact assessments were conducted to examine contingency tables. Based on dichotomized METAscore, patients were grouped into high and low METAscore groups and R package sva was employed to diminish the computational batch effect. These data were all visualized using the ggplot2 package in R. Benjamini–Hochberg method was used in the differential gene analysis to identify significant genes by converting the *p* values into FDRs (Benjamini and Hochberg, 1995). OncoPrint was applied to depict the mutation landscape of the TCGA dataset using maftools package in R (Gu et al., 2016). Cluster survival curves were generated using the Kaplan-Meier evaluation, and the log-rank test was employed to determine the differences statistical significance. Hazard ratios were computed using the univariate and multivariate Cox proportional hazards regression models. Independent prognostic factors were determined using the R survival package. Survival curves were generated using the survminer package. Heatmaps were generated using pheatmap function (https://github.com/raivokolde/pheatmap). All statistical and computational analyses were conducted using R programming (https://www.r-project.org/). These tests were two-sided and *p* value less than 0.05 signified statistical significance.

## Results

### Three Metabolism-Relevant Clusters in CESC Differ in Immune Characteristics

**FIGURE 1:**
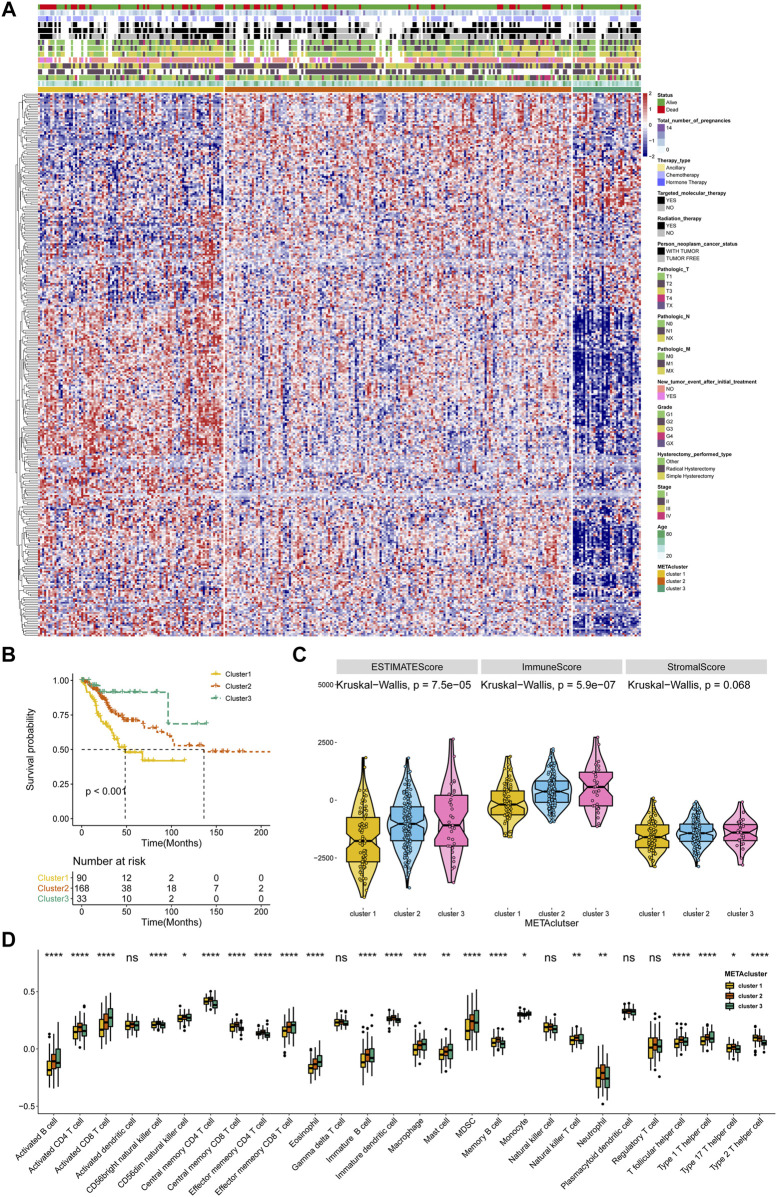
Identification of three METAclusters in the TCGA data of CESC. **(A)** Unsupervised clustering of CESC patients based on 315 identified metabolic genes: METAcluster 1 (*n* = 90), METAcluster 2 (*n* = 168) and METAcluster 3 (*n* = 33). **(B)** Kaplan–Meier curves for survival time of the three METAclusters in CESC. Log-rank test presented an overall *p* < 0.001. **(C)** A Violin plot showing ESTIMATE score, immune score and stromal score of the three METAclusters. **(D)** A Boxplot showing the abundance of immune cell populations among the three METAclusters. In each cluster, the top and bottom of the boxes represent the 75th and 25th percentiles (interquartile range), respectively, and thick line in the boxes represents median value. The statistical differences among the three METAclusters were determined using the Kruskal-Wallis test. The statistical *p* value was represented by asterisks (ns represents no significance; *p < 0.05; **p < 0.01; ***p < 0.001; ****p < 0.0001).

A flow diagram for the steps of this study was presented in Supplementary Figure S1. The 2,752 metabolic genes, encoding all human small molecule transporters and metabolic enzymes obtained from literature screening, were subjected to metabolism-related cluster classification by unsupervised clustering in the 291 CESC samples from TCGA. After assessing, a total of 315 candidate genes were sorted out, and clustering of the TCGA CESC samples was performed based on these genes using the ConsensusClusterPlus package in R. The optimal *k* number was determined as, compared with *k* = 2 or 4, the consensus matrix heatmap presented distinct and sharp boundaries when *k* = 3, supporting the robust and stable sample clustering. Thus, three distinct METAclusters were determined that 90 cases in METAcluster 1, 168 cases in METAcluster 2 and 33 cases in METAcluster 3 (Figure 1A; Supplementary Figures S2A–D). Survival analysis revealed the significant difference in patient overall survival (OS) time among these METAclusters, hinting the prognostic value in CESC (Figure 1B).

ESTIMATE is a tool using gene expression data to predict tumor purity and the presence of tumor immune/stromal cell infiltration. The ESTIMATE algorithm mainly generates three score-patterns to quantify the overall infiltration: 1) an ESTIMATE score that signifies tumor purity, 2) an immune score that infers the invasion of immune cells, and 3) a stromal score that denotes the presence of stromal cells. Significant differences in ESTIMATE and immune score, but not stromal score, were presented among the three METAclusters (Figure 1C). We next evaluated immune infiltration to describe their immune landscape. An abundance of 28 immune-correlated cell populations was computed using the ssGSEA. In accordance, result showed an obvious differential expression in immune cells (B cells, CD4^+^ T cells, CD8^+^ T cells, immature dendritic cells, macrophage, mast cell, MDSC, neutrophils, monocyte, and T helper cell) among the METAclusters. These data distinctly indicated these three METAclusters maintained different immune-relevant signatures (Figure 1D).

**FIGURE 2:**
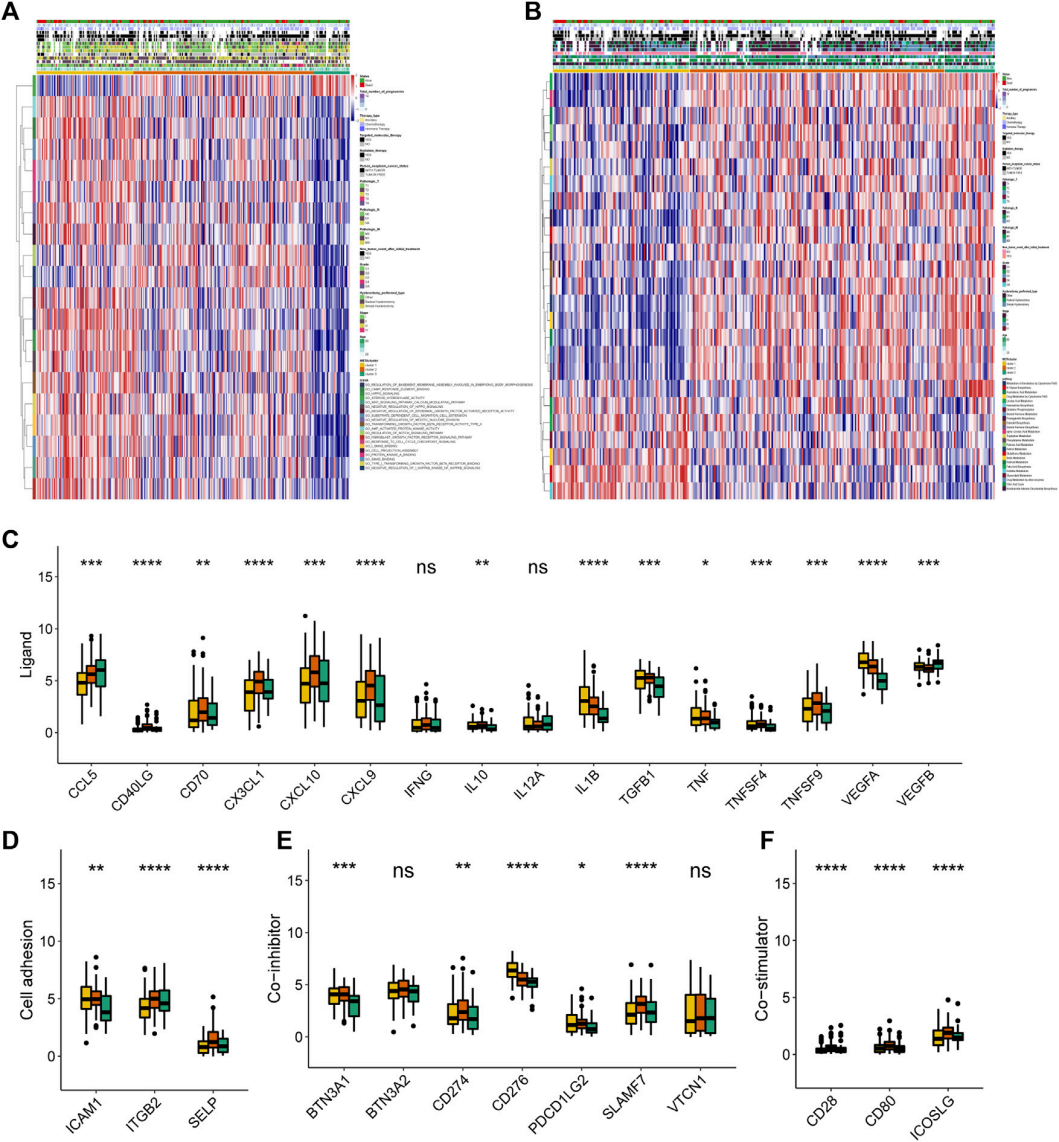
Transcriptome, metabolic and immune characteristics of the three METAclusters. **(A)** Pathway enrichment analysis showing the differential activated transcriptome pathways in each METAcluster. **(B)** Pathway enrichment analysis showing the differential activated metabolic pathways in each METAcluster. Heatmaps were plotted to visualize the biological processes. **(C–F)** Immune checkpoints expression (normalized count) in each METAcluster. The statistical *p* value was represented by asterisks (ns represents no significance; *p < 0.05; **p < 0.01; ***p < 0.001; ****p < 0.0001).

With the remarkable difference in immune characteristic identified, to further typify the transcriptomic and metabolic behavior differences among these metabolic patterns, we applied the GSVA enrichment analysis. Pathway analysis revealed that key carcinogenic activation pathways in CESC including WNT, HIPPO, NOTCH, NF-κB, and TGFβ (Maliekal et al., 2008; Ramos-Solano et al., 2015; Zhu et al., 2016; Bahrami et al., 2017; Tilborghs et al., 2017; Rodrigues et al., 2019; Wang et al., 2020) (Figure 2A), and metabolic pathways (Figure 2B) were differentially activated among these METAclusters which emphasized the genetic significance of the metabolism-based classification.

Then subsequent analysis investigated the expression of key immune checkpoints which have been selected based on current clinical trials drug inhibitors in other specific cancer types. As shown, this analysis revealed discriminable expression in ligand, cell adhesion, co-inhibitor, co-stimulator, antigen present, receptor and other checkpoints (Figures 2C–F, Supplementary Figures S2E–G). Considering these immune checkpoints were in charge of regulating the immune activation through modulating the T-cell in the immune respond process, we inferred that in CESC, respective METAcluster possessed different immune checkpoint blockade efficacy presumably.

### Validation Performance of the Metabolism-Based Classification

**FIGURE 3:**
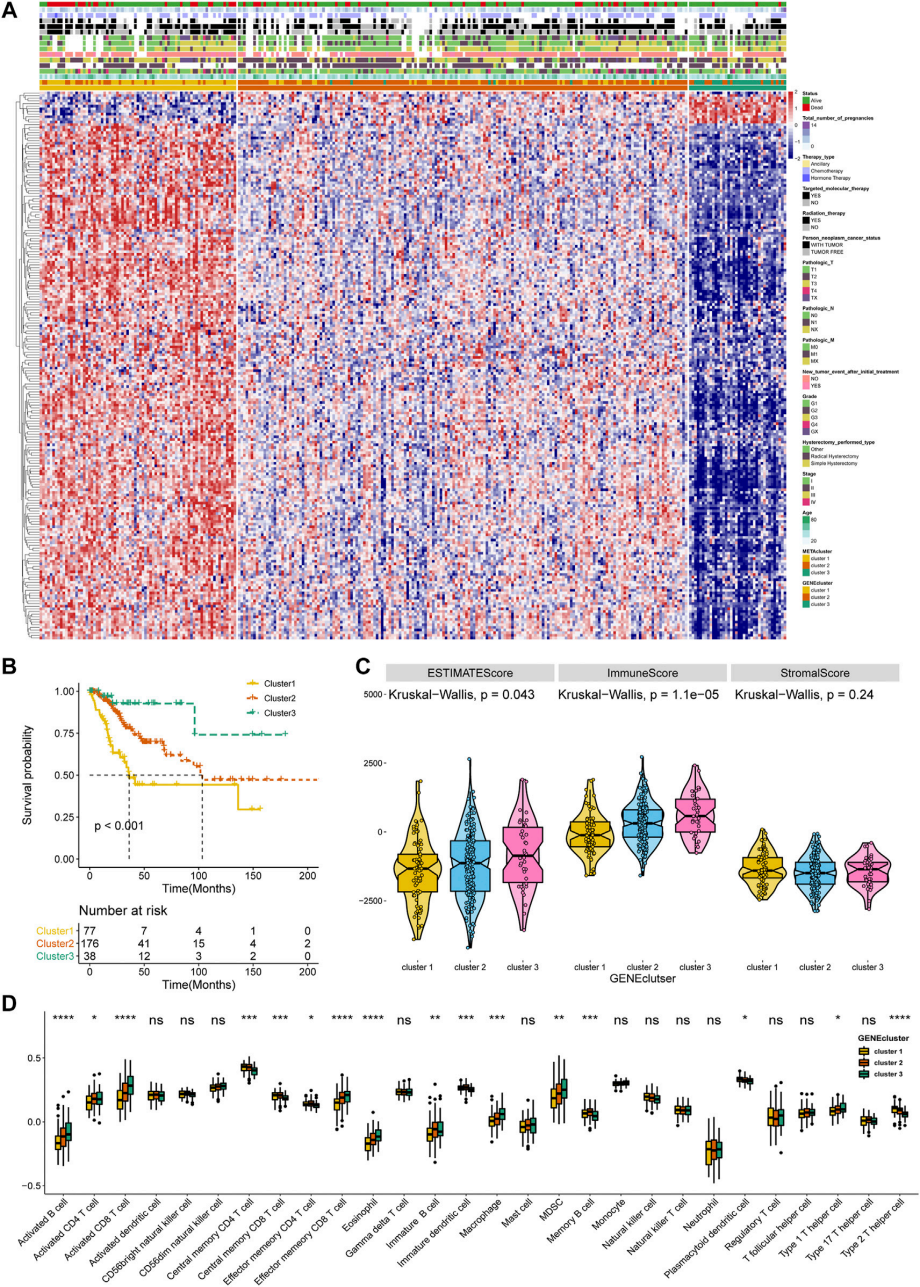
Identification of CESC geneclusters based on DEGs of METAclusters. **(A)** Unsupervised clustering of CESC patients on 207 identified DGEs: genecluster 1 (*n* = 77), genecluster 2 (*n* = 176), and genecluster 3 (*n* = 38). **(B)** Kaplan–Meier curves for survival time of the three geneclusters in CESC. Log-rank test presented an overall *p* < 0.001. **(C)** A Violin plot showing ESTIMATE score, immune score and stromal score of the three geneclusters. **(D)** A Boxplot showing the abundance of immune cell populations in the three geneclusters. The statistical differences among the three geneclusters were determined using the Kruskal-Wallis test. The statistical *p* value was represented by asterisks (ns represents no significance; *p < 0.05; **p < 0.01; ***p < 0.001; ****p < 0.0001).

**FIGURE 4:**
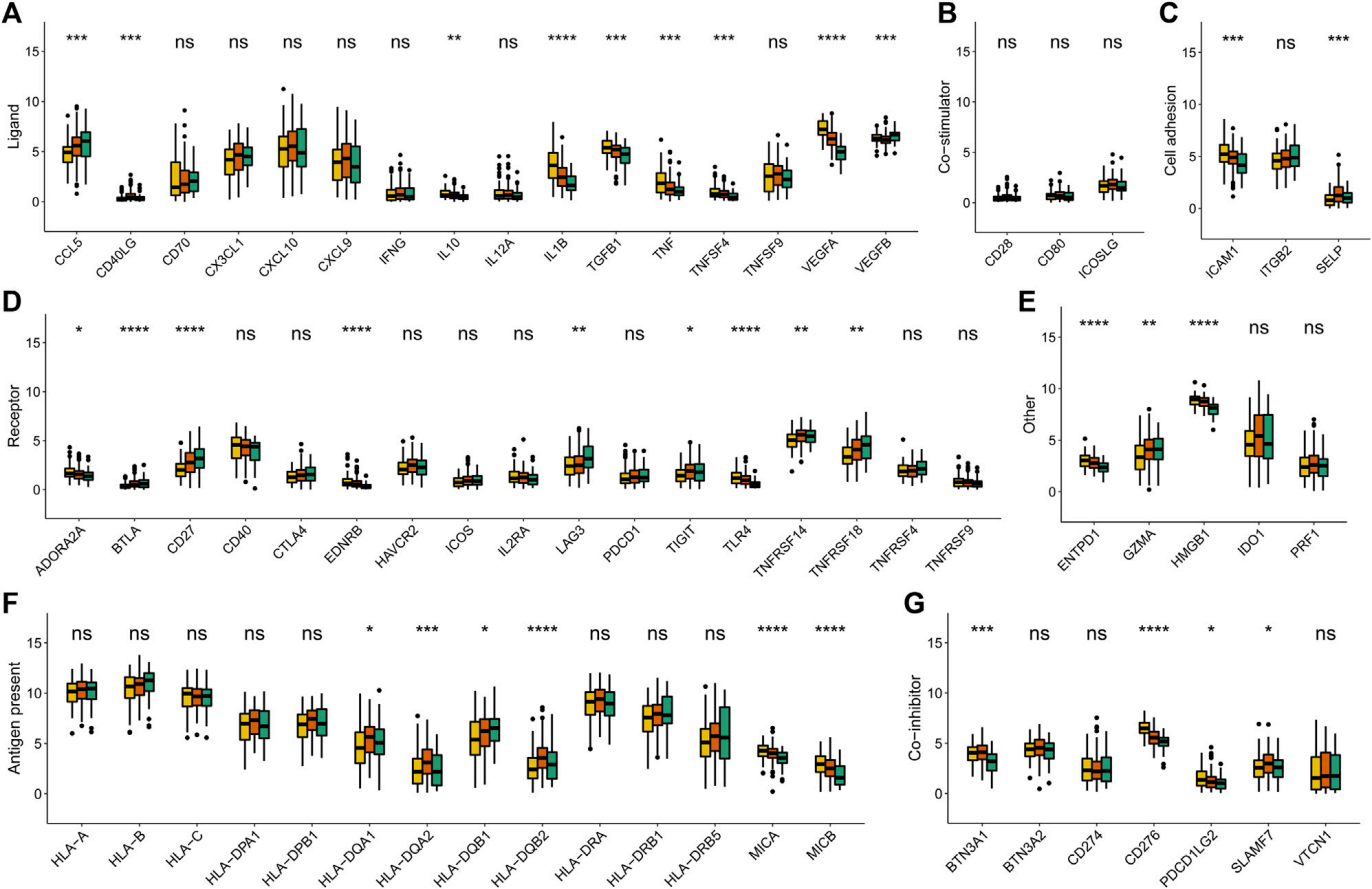
Immune checkpoints expression (normalized count) in each genecluster. The statistical differences were determined using the Kruskal–Wallis test and the statistical *p* value was represented by asterisks (ns represents no significance; *p < 0.05; **p < 0.01; ***p < 0.001; ****p < 0.0001).

To affirm metabolism-phenotype distinction of each METAcluster, unsupervised cluster analysis of 207 most representative DEGs among three METAclusters obtained using the limma package (Smyth, 2004) was completed to separate CESC patients into genomic subtypes (Figure 3A). The optimal cluster number supported the existence of three distinct and robust geneclusters in CESC patients (Supplementary Figure S3). Among these three geneclusters, the prominent difference in OS was strikingly consistent with the result of METAclusters (Figure 3B). Also, the expression of ESTIMATE and immune scores (Figure 3C), immune infiltration (Figure 3D), as well as the key immune checkpoints expression (Figure 4) were all highly in accordance with the differences among the METAclusters, genomically verifying three distinct metabolism-associated patterns in CESC.

### METAscore Generation and Characteristics

**FIGURE 5:**
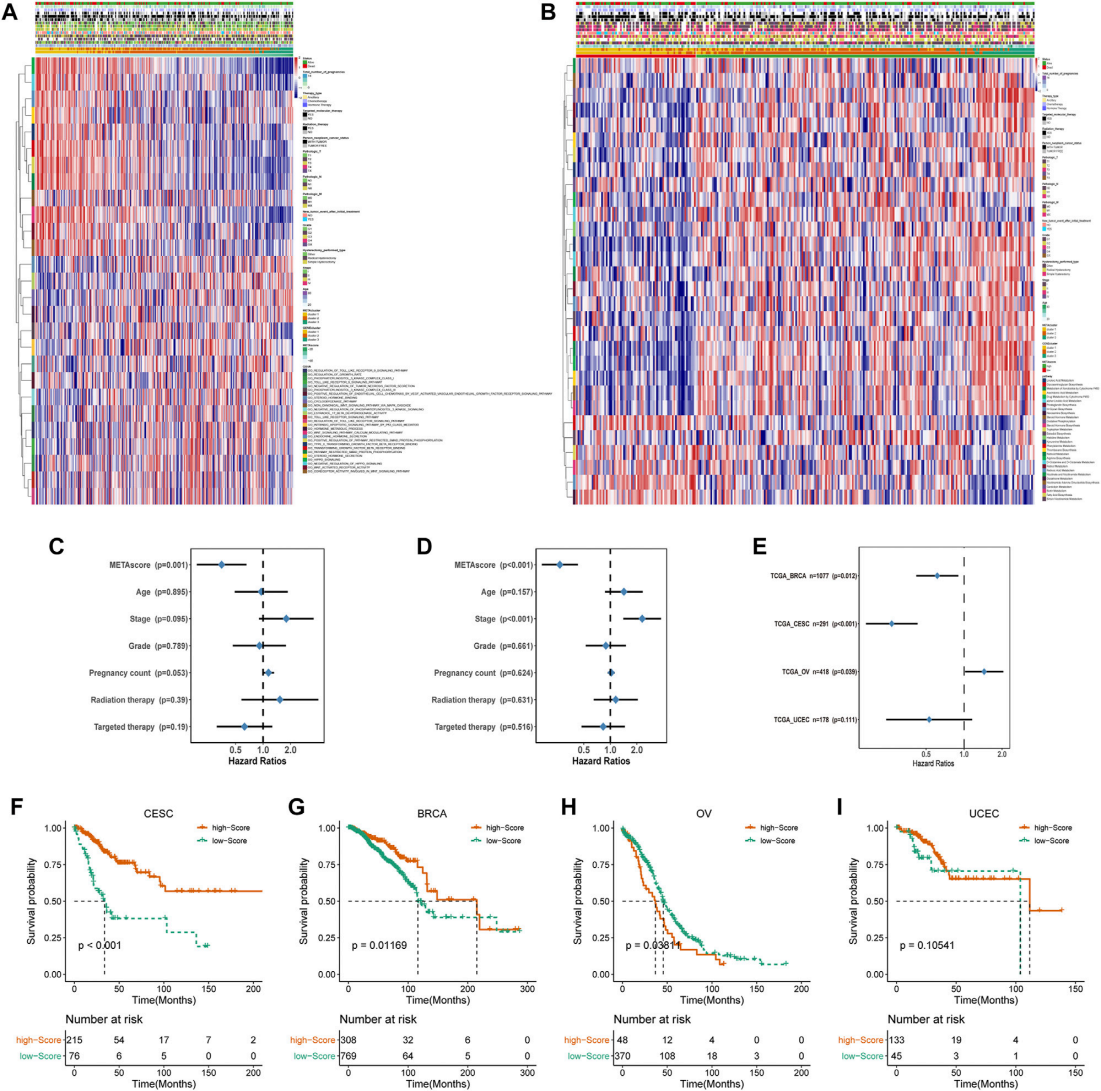
Construction of METAscore and functional annotation. **(A)** Pathway enrichment analysis showing the differential activated transcriptome pathways related to METAscore. **(B)** Pathway enrichment analysis showing the differential activated metabolic pathways related to METAscore. Heatmaps were plotted to visualize the biological processes. **(C–D)** Hazard ratios (HR) of METAscore in multivariate **(C)** and univariate **(D)** cox regression models combined with CESC patient clinical characteristics. **(E)** Hazard ratios (HR) of METAscore estimating the prognostic value in different gynecologic cancers. The horizontal line length represents the 95% confidence interval for each cancer type. The vertical line represents HR = 1. **(F-I)** Kaplan–Meier curves for survival of the high- and low- METAscore groups in CESC (**(F)**, *p* < 0.001, log-rank test), BRCA (**(G)**, *p* = 0.01169, log-rank test), OV (**(H)**, *p* = 0.03811, log-rank test). and UCEC (**(I)**, *p* = 0.10541, log-rank test).

Given the individual complexity and heterogeneity of metabolic modification, we used the PCA algorithm to construct the METAscore to quantify metabolic patterns in CESC patients. Two aggregate score groups (high- and low- METAscore groups) were generated by the sum of relevant scores, and GSVA analysis uncovered that the METAscore was related to the immune signaling pathways, cancer pathways (Figure 5A), and key metabolic pathways (Figure 5B).

Then we evaluated the potential of the METAscore to predict CESC survival. Univariate and multivariate Cox regression model analysis, which considered including patient age, stage, grade, pregnancy count, radiation therapy and targeted therapy, confirmed that the METAscore was an independent and reliable prognostic biomarker (Figures 5C,D). Besides, the prognostic significance of the METAscore was measured in four independent gynecological cancers including CESC, breast cancer (BRCA), ovarian cancer (OV) and endometrial cancer (UCEC) (Figure 5E). Notable OS differences emerged between the high- and low-METAscore groups in BRCA, OV and CESC which were all recognized as hot tumors with distinct T-cell invasion (Figures 5F–I).

**FIGURE 6:**
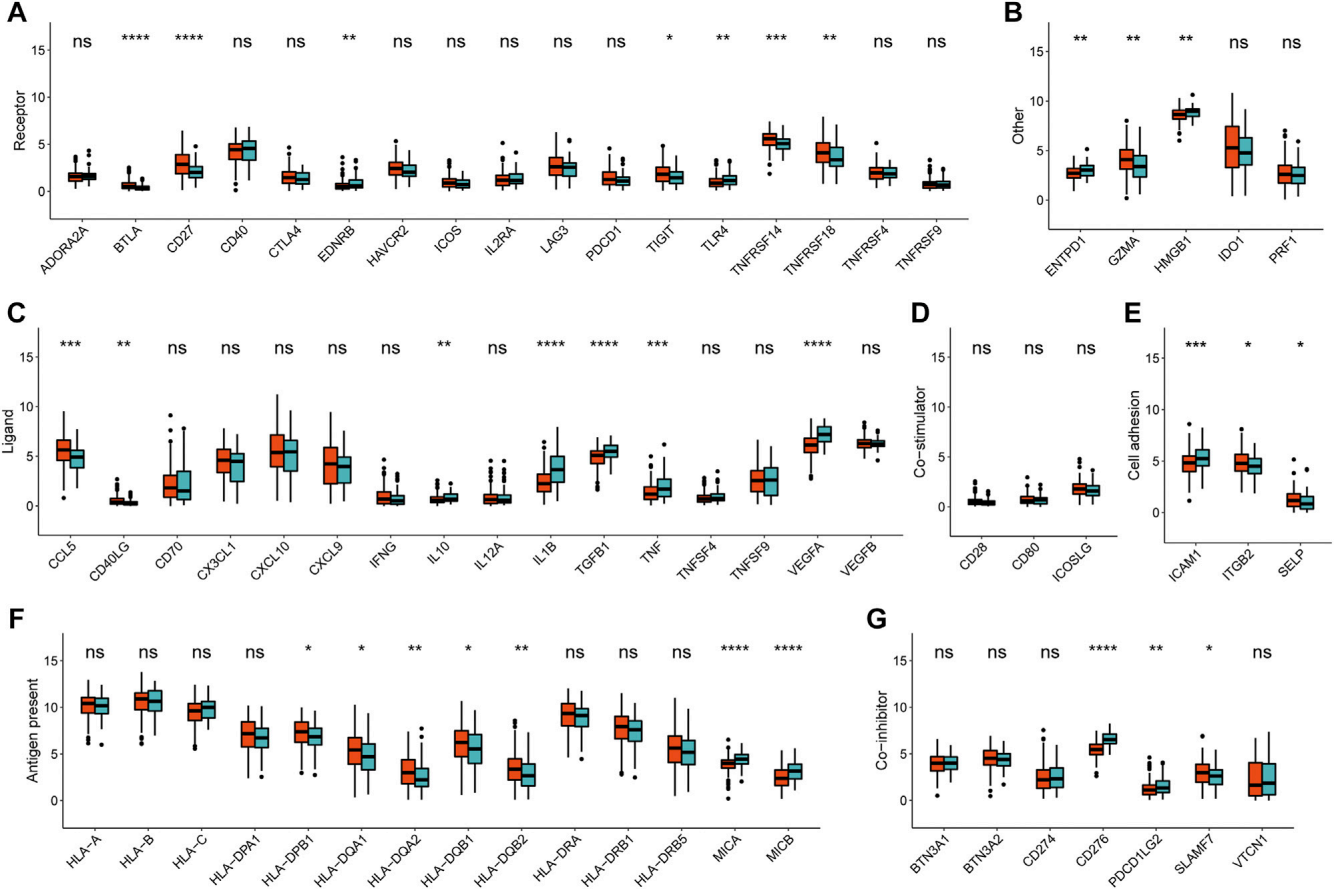
Immune checkpoints expression (normalized count) between high- and low- METAscore groups of CESC patients. The statistical differences were determined using the Kruskal–Wallis test and the statistical *p* value was represented by asterisks (ns represents no significance; *p < 0.05; **p < 0.01; ***p < 0.001; ****p < 0.0001).

Accordingly, the next evaluation was concerned in immune checkpoints expression between two METAscore groups. Robust correlation between METAscore and different response of immune checkpoints including receptor, ligand, cell adhesion, co-inhibitor, antigen present and other checkpoints was demonstrated, indicating the guiding role in immunotherapy of METAscore (Figure 6).

### Correlation Between METAscore and CESC Genomic Signatures

**FIGURE 7:**
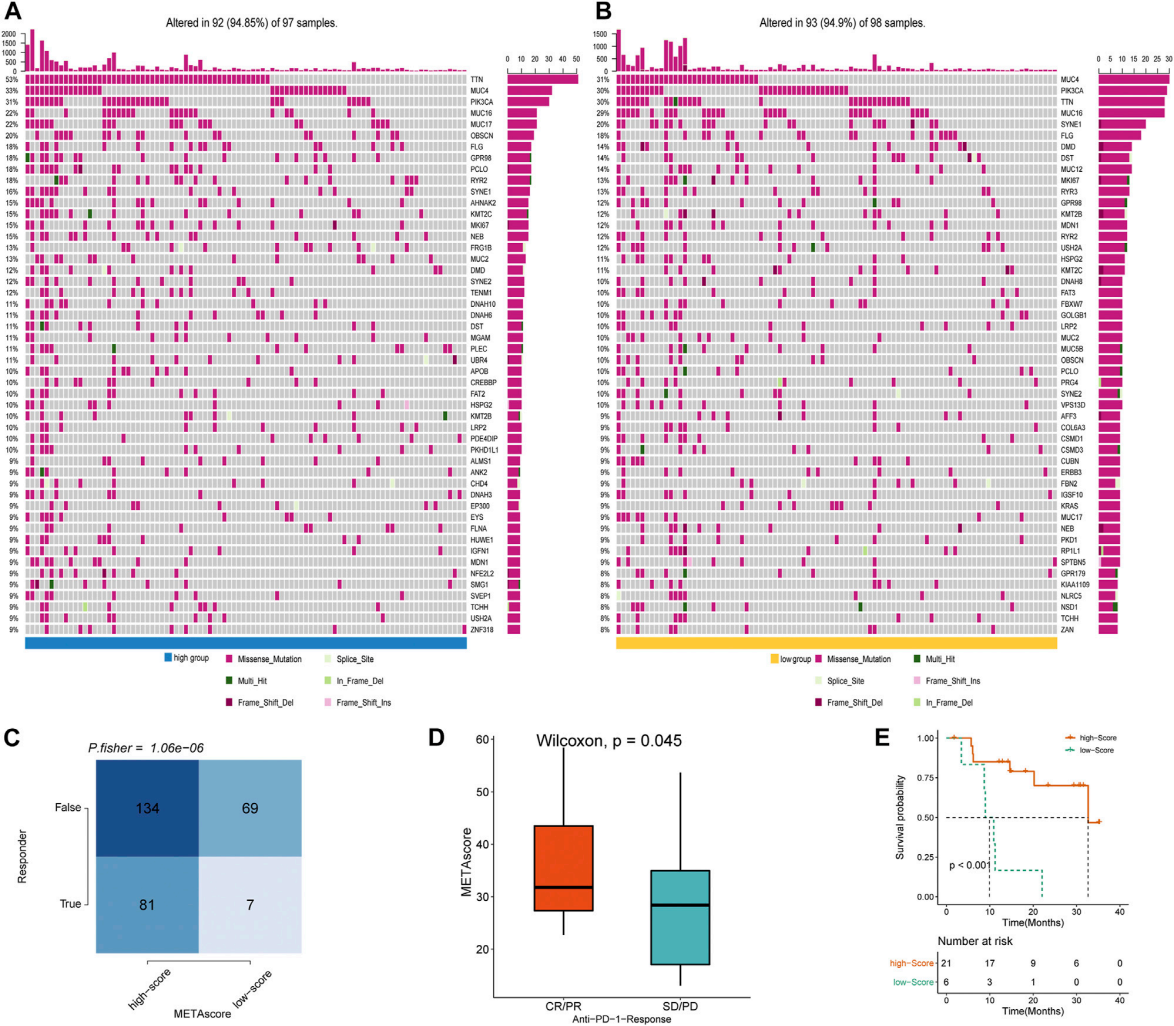
METAscore predicts immunotherapeutic benefit. **(A–B)** The oncoPrint established by CESC patients with high-METAscore **(A)** and low- METAscore **(B)**. **(C)** TIDE prediction between high- and low- METAscore group. **(D)** Rate of clinical response (complete response [CR]/partial response [PR] and stable disease [SD]/progressive disease [PD]) to anti–PD-L1 immunotherapy in high- or low- METAscore groups in the GSE78220 cohort. **(E)** Kaplan–Meier curves for survival of patients with high- (*n* = 21) and low- (*n* = 6) METAscore in the GSE78220 cohort. Log-rank test presented an overall *p* < 0.001.

To determine the difference in somatic mutation frequencies between the high- and low- METAscore groups, we analyzed the TCGA genomic files. The consequence revealed that the high- and low- METAscore groups exhibited distinct mutation characteristics and the genes with a high mutation frequency in TTN, MUC4, PIK3CA, and MUC16 which all correlated with EMT (Chen et al., 2018) and critical cancer pathways including PI3K/AKT (Razia et al., 2019) and JAK2/STAT3 (Shen et al., 2020) (Figures 7A,B) in CESC. Somatic mutations in the PIK3CA denoted a late event during cervical carcinogenesis, and have been detected in multiple cervical carcinoma subgroups (Verlaat et al., 2015). Besides, MUC4 and PIK3CA were frequently mutated in the HPV16-KRT, a HPV16 subtype typified by increased expression of keratinization genes, biological oxidation and Wnt pathway signaling (Lu et al., 2019). Similarly, regarding altered somatic copy number variation (CNV), remarkable differences in driver gene amplification and deletion were emerged between the METAscore groups (Supplementary Figure S4). These analyses revealed a high genomic heterogeneity and altered gene expression landscape during the METAscore groups, supporting the association between the METAscore and genomic alterations.

### METAscore Predicts Immunotherapeutic Benefits

Immune checkpoint blockade (ICB) therapy is a revolutionary immune-based treatment in cancers including CESC. Inhibition of the immune checkpoints using monoclonal antibodies that block the T-cell molecules PD-1, PD-L1, as well as CTLA4 has emerged as a novel anti-cancer treatment with extraordinary survival advantages (Curran et al., 2010). Considering the correlation between the METAscore and immune characteristics, we elaborated the predictive potential of the METAscore to estimate ICB therapeutic value by using the melanoma GSE78220 cohort. TIDE algorithm is a method of modeling two primary mechanisms of tumor immune infiltration: the stimulation of T-cell dysfunction companying with high cytotoxic T-lymphocytes (CTL) infiltration, and the prevention of T-cell infiltration with low CTL levels, which estimates potential response to immunotherapy (Wang et al., 2020b). We conducted TIDE algorithm and obtained that patients in high- METAscore group tended to respond to immunotherapy, prompting CESC patients with high- METAscore might more likely benefit from immunotherapy (Figures 7C,D). Combined with prediction of survival outcomes in CESC (Figure 7E), we assured the guiding value of METAscore in immunotherapy.

## Discussion

Although new CESC classification systems hinged on gene expression and imaging have been anticipated currently, it has not reached a molecular taxonomic consensus yet. Emerging evidence supported that the metabolism dysfunction acted a pivotal part in CESC proliferation and metastasis. Our study innovatively proposed a metabolism-relevant classification which classified the CESC patients into three METAclusters, as exhibited distinct differences in patient survival outcomes, metabolic signatures, immune signatures, genomic signatures and immunotherapy efficiency. Then, METAscore, a scoring system designed to evaluate the CESC patient comprehensive metabolic-pattern and related to genetic alteration, was an independent prognostic biomarker for estimating survival result and an immunotherapy predictive indicator for guiding more precise therapy in CESC. What should be of concern is our study revealed the comprehensive landscape of interactions between the metabolic signature and immune phenotypes in CESC.

The CESC microenvironment consist of immune-suppressive cells, as well as high expression of immune checkpoint biomolecules. Immune evasion by tumor cells, T-cell exhaustion and tumor-specific T-cell dysfunction are all the results of the contact between PD-1 and PD-L1 on tumor cells and tumor-infiltrating lymphocytes (Wherry and Kurachi, 2015). Researchers supported that immune dysfunction had a great repercussion in CESC progression and patient clinical outcome (Carvalho et al., 2016; de Vos van Steenwijk et al., 2013). As a fresh area in oncology, immunometabolism is a burgeoning branch dealing that interfaces immune function with metabolic modulation. The dynamism of the immune system augments the tumor microenvironment complexity, as various immune populations and metabolic pathways often interfere mutually (Wang et al., 2019). Combined with previous published findings (Dugnani et al., 2017; Domblides et al., 2018), our data adds the evidence of the complex interplay between the metabolism and immune function in CESC.

Recently, cancer immunotherapy has gained widespread attention. The mounting successes of immune-based treatments for solid tumors have spurred numerous cancer clinical trials testing strategies to elicit tumor-specific immune responses, either alone, in combination with ICB, or with traditional cancer therapies. Among, the PD-1/PD-L1 pathway has received significant consideration because of its function on eliciting T-cell immune checkpoint responses which results in immune surveillance evasion of tumor cells and resistance to chemotherapy. Hence, the application of anti-PD-1/PD-L1 antibodies as checkpoint inhibitors has rapidly became a prospective anti-cancer strategy. Analysis of the efficacy and safety of the PD-1 immune checkpoint inhibitors has offered promising results in the past few years (Davis and Patel, 2019; Wang and Li, 2019). Intriguingly, the immune checkpoints have emerging positions in modulating the metabolic activity of T cells. Moreover, recent investigations on cancer metabolism have disclosed that the dysregulated metabolic activity of tumour-infiltrating immune cells and tumour cells contribute to the impaired antitumour immune responses in the TME (Li et al., 2019). Our observation that distinct expression of immune checkpoint genes in three METAclusters, raised that CESC patients in different subclusters maintained varying degrees of immunotherapy treatment significance, which hinted the association between the CESC metabolic signatures and guiding significance for cancer immunotherapy.

Yet, as one of the most promising breakthroughs, ICB immunotherapy is only beneficial in a small proportion of cancer patients, ostensibly owing to insufficient immunosuppressive tumour microenvironment (TME) reprogramming and consequently limited reinvigoration of anti-tumor immunity. Multiple studies have shown that PD-1, as well as PD-L1 expression and mutation load, are not efficient to mirror ICB aids (Roh et al., 2017; Fuchs et al., 2018). Development of novel biomarkers for checkpoint immunotherapy responses is imperative for improving the therapeutic outcomes (Hugo et al., 2016; Nishino et al., 2017; Panda et al., 2018). Felicitously, the METAscore performed as a predictive biomarker for CESC prognosis in this study.

Moreover, the genetic mutations in cancerous tissues are often disrupted accompanied by metabolic harmony. Previous preclinical (Burr et al., 2017) and clinical (George et al., 2017) reports have exposed the influence of the genetic heterogeneity on ICB responses, presumably as overall mutation load drove T-cell responses (Diaz and Le, 2015; McGranahan et al., 2016). Our data suggested that METAscore was correlated with the genomic mutational load and CNV, promoting that METAscore could delegate the dynamic of immunometabolism from the genetic aspect.

Therefrom, we confirmed immunotherapy treatment effective and survival outcome discrepancy between the two METAscore groups, which was a compelling clue that METAscore could evaluate the sensitivity to antitumor immunotherapy. Incumbent data on the scoring system and the prognostic scores of CESC mainly concentrated on the perspectives of immunogenomics and genetic alteration (Cai et al., 2020; Li et al., 2019; Wang et al., 2021; Zhang et al., 2021) . Comparatively, the METAscore developed in our study was a promising breakthrough on the immunometabolism, offering novel insights into CESC immune diversity from the metabolic landscape and highlighting that METAscore could predict sensitivity to immunotherapy. Taking the METAscore into consideration in the choice of comprehensive anticancer treatment might improve patient survival result. However, to maximize immunotherapeutic benefits, more clinical and tumor microenvironmental factors should be integrated into the estimation model. Next step we will explore the anchors between metabolic circuits and antitumour immunity, and the possible methods to target these pathways in the aspect of immunotherapy.

## Data Availability

The original contributions presented in the study are included in the article/Supplementary Material, further inquiries can be directed to the corresponding author.

## Supplementary Material

Supplementary Figure 1 A flow diagram for the steps of this study.

Supplementary Figure 2 The clustering map of metabolic genes and immune checkpoints expression (normalized count) in each METAcluster.

Supplementary Figure 3 The clustering map of DEGs among the three METAclusters.

Supplementary Figure 4 Status of CNV in high- and low- METAscore groups of CESC patients.
