# Supplementary figures and images for: Metabolism-Relevant Molecular Classification Identifies Tumor Immune Microenvironment Characterization and Immunotherapeutic Effect in Cervical Cancer
Source: Front Mol Biosci. 2021 Jul 1;8:624951. doi: 10.3389/fmolb.2021.624951 (PMC8280349; doi:10.3389/fmolb.2021.624951)

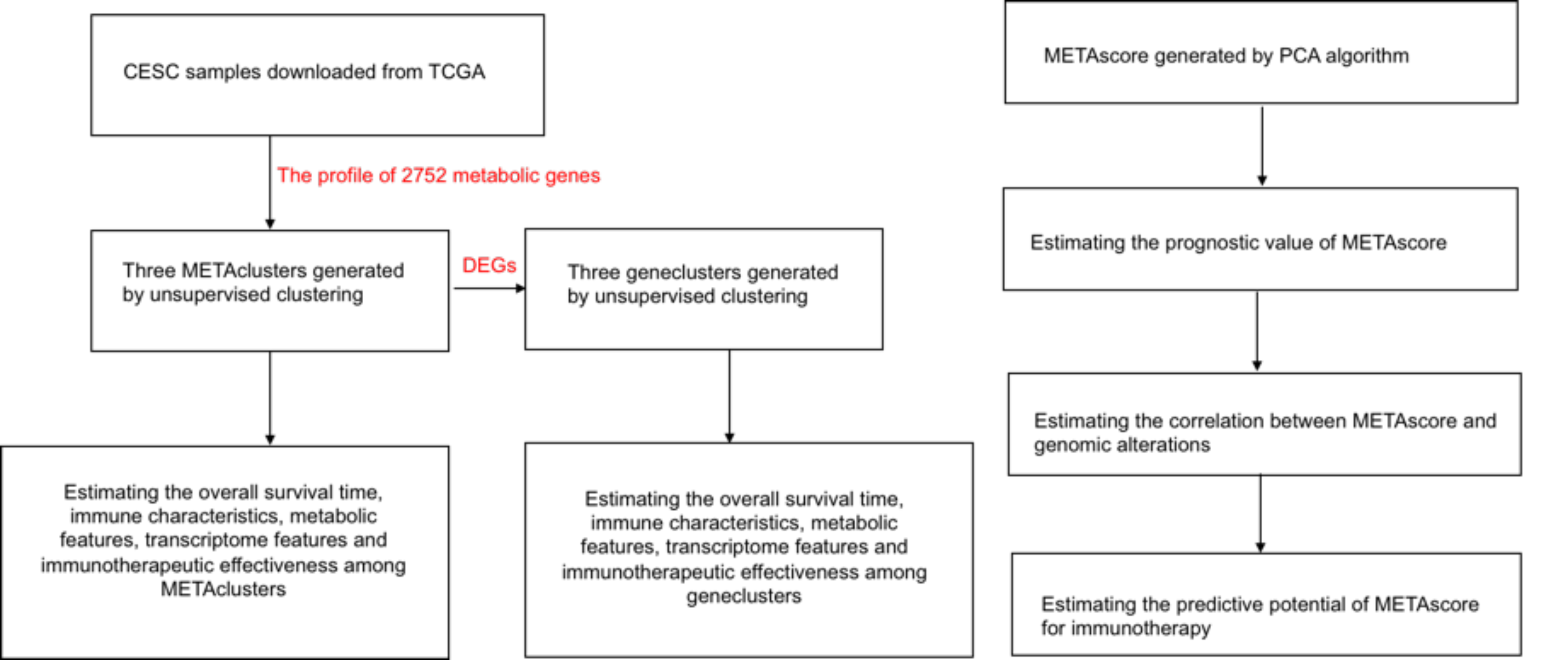

Supplement: Supplementary file 1 [file Image1.TIF]

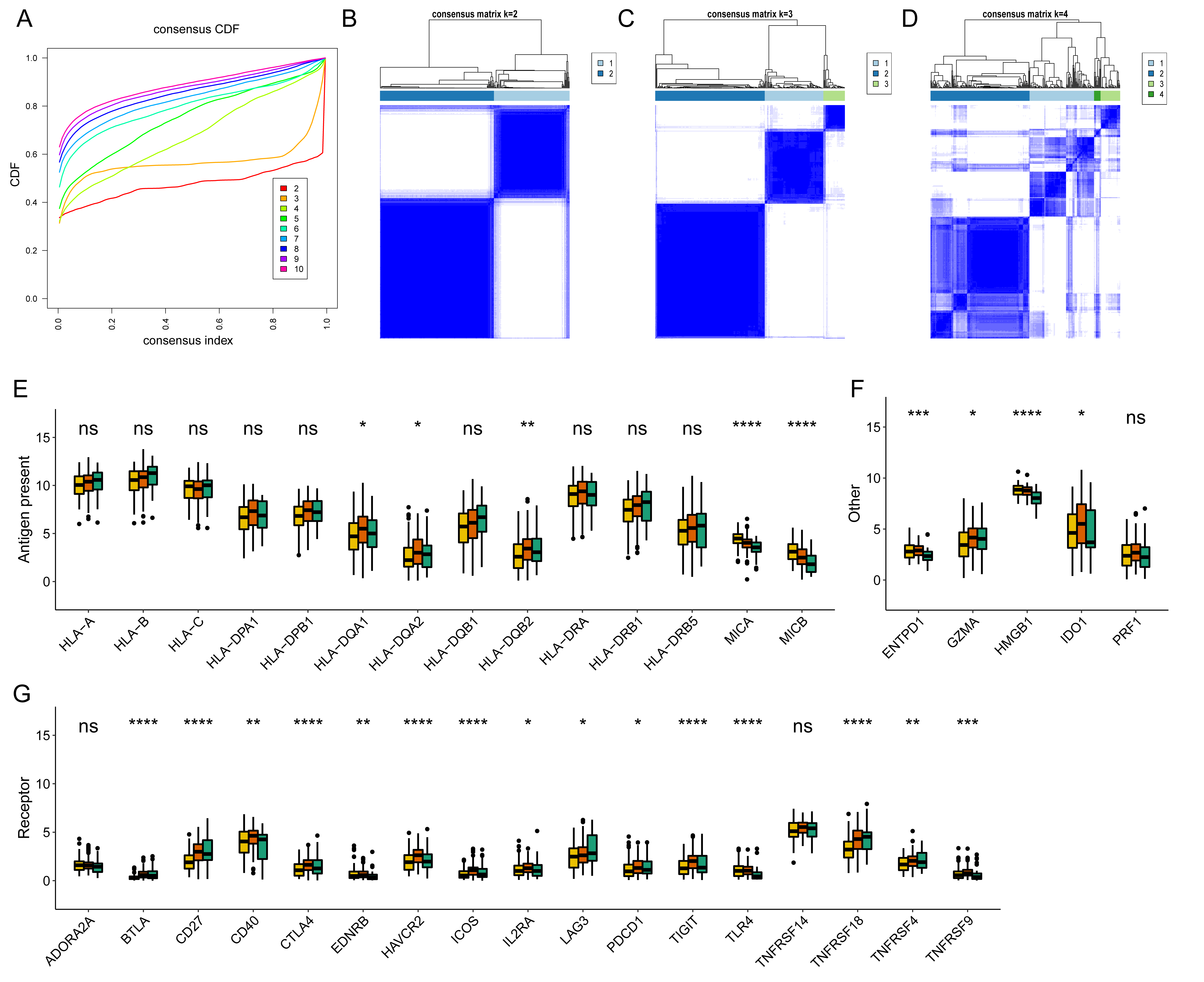

Supplement: Supplementary file 2 [file Image2.TIF]

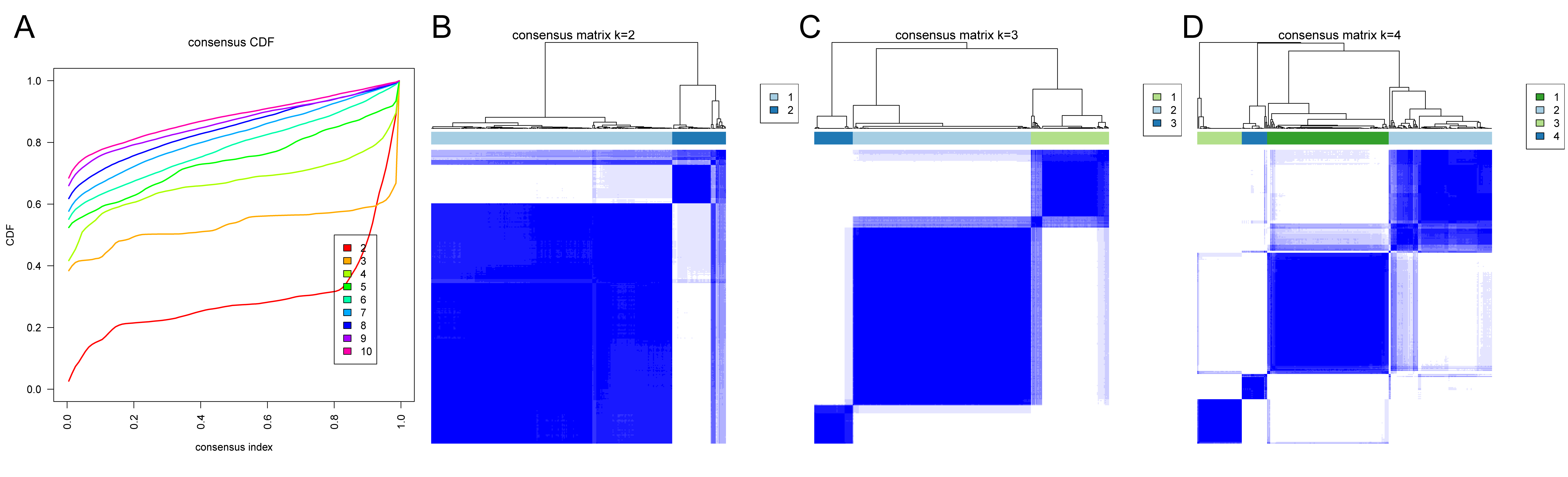

Supplement: Supplementary file 3 [file Image3.TIF]

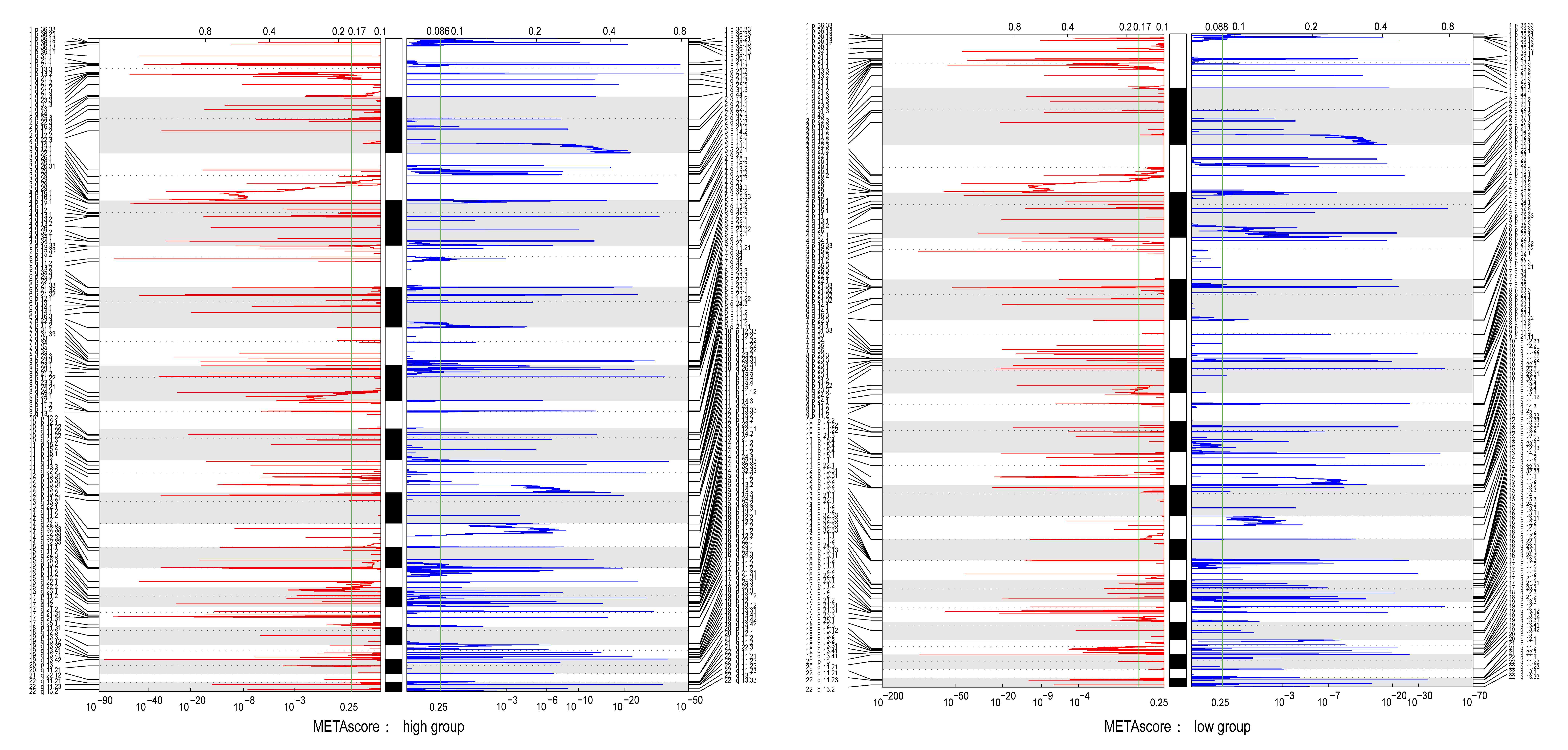

Supplement: Supplementary file 4 [file Image4.tif]
